# Supplementary material for: Functional interactions between anteroventral periventricular kisspeptin neurons and gonadotropin‐releasing hormone neurons in female mice
Source: J Neuroendocrinol. 2026 Oct 5;38(10):e70281. doi: 10.1111/jne.70281 (PMC13638252; doi:10.1111/jne.70281)
Supplement: Supplementary file 1 [file JNE-38-e70281-s002.pdf]

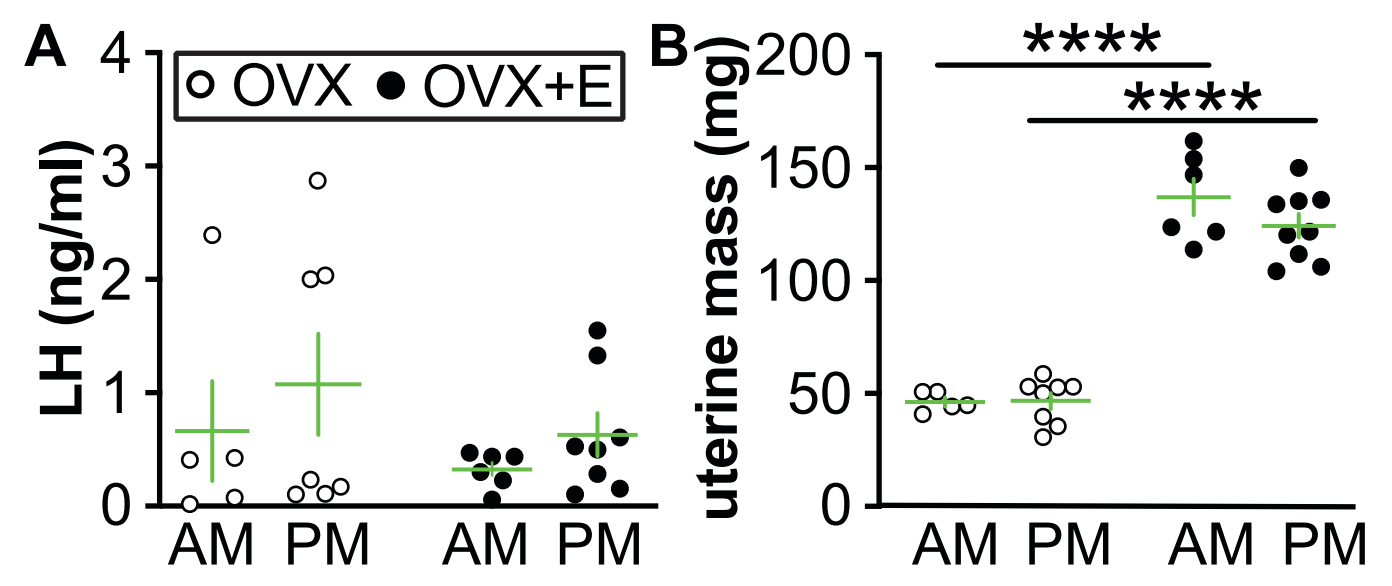

A
OVX
OVX+E
LH (ng/ml)
0
1
2
3
4
AM
PM
AM
PM
B
uterine mass (mg)
0
50
100
150
200
****
****
AM
PM
AM
PM
