# Supplementary material for: Functional interactions between anteroventral periventricular kisspeptin neurons and gonadotropin‐releasing hormone neurons in female mice
Source: J Neuroendocrinol. 2026 Oct 5;38(10):e70281. doi: 10.1111/jne.70281 (PMC13638252; doi:10.1111/jne.70281)
Supplement: Supplementary file 2 [file JNE-38-e70281-s003.pdf]

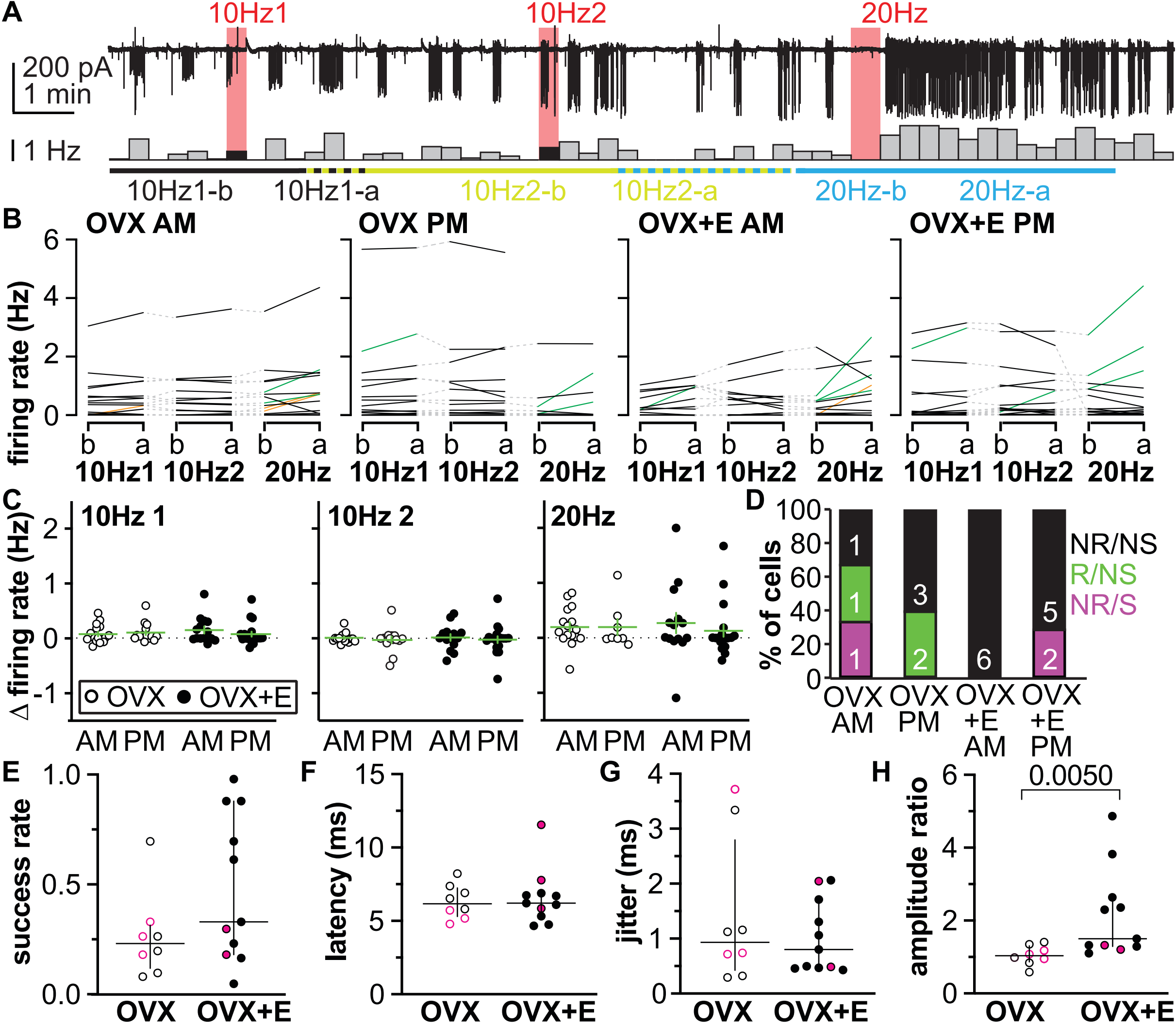

A
10Hz1
10Hz2
20Hz
200 pA
1 min
1 Hz
10Hz1-b
10Hz1-a
10Hz2-b
10Hz2-a
20Hz-b
20Hz-a
B
OVX AM
OVX PM
OVX+E AM
OVX+E PM
firing rate (Hz)
b a b a b a
10Hz1 10Hz2 20Hz
C
10Hz 1
10Hz 2
20Hz
Δ firing rate (Hz)
OVX
OVX+E
AM PM AM PM
D
% of cells
NR/NS
R/NS
NR/S
OVX AM
OVX PM
OVX +E AM
OVX +E PM
E
success rate
F
latency (ms)
G
jitter (ms)
H
amplitude ratio
0.0050
OVX
OVX+E
