# Supplementary material for: Functional interactions between anteroventral periventricular kisspeptin neurons and gonadotropin‐releasing hormone neurons in female mice
Source: J Neuroendocrinol. 2026 Oct 5;38(10):e70281. doi: 10.1111/jne.70281 (PMC13638252; doi:10.1111/jne.70281)
Supplement: Supplementary file 3 [file JNE-38-e70281-s001.docx]

**Supplementary** **Table 1** Sample sizes and statistical test results for spike fidelity and blue-exposure experiments in Figure 2. Bold text marks *P*<0.05.

| **Pulse width (all frequencies)** | **n cells, n animals** |
| --- | --- |
| 0.5 ms | (12,3) |
| 1 ms | (12,3) |
| 2 ms | (11,3) |
| *spike fidelity* |  |
| **two-way repeated-measures ANOVA** |  |
| frequency | F (2.284, 73.08) = 69.34, *P***<0.0001** |
| width | F (2, 32) = 0.8867, p=0.4219 |
| frequency x width | F (10, 160) = 0.6664, p=0.7543 |
|  |  |
| *blue exposure test - spike fidelity* | **(n cells, n animals)** |
| before | (11,3) |
| after | (11,3) |
| **Paired, two-tailed t-test** |  |
| before vs after | Diff=0.9545, CI=-0.5208 to 2.430, t=1.442, df=10, p=0.18 |

**Supplementary** **Table 2.** Two-way ANOVA analysis of uterine mass and LH in the daily surge model

| **Test / Source of Variation** | **F (DFn, DFd)** | **P value** |
| --- | --- | --- |
| **I. Uterine Mass** |  |  |
| time of day | F(1, 24) = 1.29 | 0.2667 |
| estradiol | F(1, 24) = 244.7 | < 0.0001*** |
| time of day x estradiol | F(1, 24) = 1.47 | 0.2374 |
| **Post-hoc: estradiol** | **Mean Diff.** | **Adjusted *P*** |
| AM: OVX vs. OVX+E | -90.65 | < 0.0001**** |
| PM: OVX vs. OVX+E | -77.61 | < 0.0001**** |
| **Post-hoc: time** |  |  |
| OVX: AM vs. PM | -0.40 | 0.9984 |
| OVX+E: AM vs. PM | 12.63 | 0.1836 |
| **II. LH** |  | **P value** |
| time of day | F(1, 22) = 1.25 | 0.2762 |
| estradiol | F(1, 22) = 1.49 | 0.2357 |
| time of day x estradiol | F(1, 22) = 0.02 | 0.8773 |

**Supplementary** **Table 3.** Three-way mixed-effects model analysis of baseline firing rate in the daily surge model.

| **Source of Variation** | **F (DFn, DFd)** | **P value** |
| --- | --- | --- |
| **stimulus** | *F*(1.08, 60.47) = 2.03 | 0.1588 |
| **time of day** | *F*(1, 59) = 0.84 | 0.3625 |
| **estradiol** | *F*(1, 59) = 2.08 | 0.1544 |
| **stim x time of day** | *F*(1.08, 60.47) = 0.72 | 0.4095 |
| **stim x estradiol** | *F*(1.08, 60.47) = 0.24 | 0.6414 |
| **time x estradiol** | *F*(1, 59) = 0.40 | 0.5316 |
| **stim x time of day x estradiol** | *F*(1.08, 60.47) = 0.21 | 0.6699 |

**Supplementary** **Table 4.** Two-way ANOVA analysis of change in firing rate in the daily surge model.

| **Frequency** | **Source of Variation** | **F (DFn, DFd)** | **P value** |
| --- | --- | --- | --- |
| **10 Hz (1)** | time of day | F(1, 59) = 0.24 | 0.6274 |
|  | estradiol | F(1, 59) = 0.19 | 0.6633 |
|  | time of day x estradiol | F(1, 59) = 1.05 | 0.3106 |
| **10 Hz (2)** | time of day | F(1, 59) = 0.40 | 0.5291 |
|  | estradiol | F(1, 59) = 0.01 | 0.9171 |
|  | time of day x estradiol | F(1, 59) < 0.01 | 0.9959 |
| **20 Hz** | time of day | F(1, 53) = 0.25 | 0.6202 |
|  | estradiol | F(1, 53) < 0.01 | 0.9905 |
|  | time of day x estradiol | F(1, 53) = 0.25 | 0.6211 |

**Supplementary** **Table 5.** Mann-Whitney U test of evoked PSC characteristics in cells from OVX vs OVX+E mice in the daily surge model.

| **Measurement** | **OVX Median (IQR) (n=8)** | **OVX+E Median (IQR) (n=11)** | **Mann-Whitney U** | **P value** |
| --- | --- | --- | --- | --- |
| **success rate (%)** | 23.33 (19.59) | 33.33 (70.00) | 28.50 | 0.2124 |
| **latency (ms)** | 6.18 (1.95) | 6.22 (1.57) | 44.00 | > 0.9999 |
| **jitter (ms)** | 0.94 (2.37) | 0.81 (1.24) | 42.00 | 0.9039 |
| **amplitude ratio** | 1.04 (0.44) | 1.50 (1.35) | 11.00 | 0.0046** |

**Supplementary** **Table 6.** Analysis of uterine mass and LH in diestrous, proestrous, and OVX (1wk) mice.

| **Test / Source of Variation** | **Statistic (H or Z)** | **df** | **Adjusted P value** |
| --- | --- | --- | --- |
| **I. Uterine Mass** |  |  |  |
| **Kruskal-Wallis Test** | **29.75** (H) | **2** | **< 0.0001****** |
| diestrus vs. proestrus | 3.317 (Z) | — | 0.0027** |
| diestrus vs. OVX (1wk) | 2.729 (Z) | — | 0.0190* |
| proestrus vs. OVX (1wk) | 5.421 (Z) | — | < 0.0001**** |
| **II. Plasma LH** |  |  |  |
| **Kruskal-Wallis Test** | **15.74** (H) | **2** | **0.0004***** |
| diestrus vs. proestrus | 3.681 (Z) | — | 0.0007*** |
| diestrus vs. OVX (1wk) | 2.777 (Z) | — | 0.0165* |
| proestrus vs. OVX (1wk) | 0.634 (Z) | — | > 0.9999 |

**Supplementary** **Table 7.** Effects of stimulation frequency and hormone state (diestrous, proestrous, OVX [1wk]) on change in firing rate.

| **Test / Source of Variation** | **F (DFn, DFd)** | **P value** |
| --- | --- | --- |
| **Two-way RM ANOVA** |  |  |
| stimulus frequency | F(2, 84) = 39.54 | < 0.0001*** |
| cycle stage | F(2, 42) = 2.42 | 0.1014 |
| stimulus x cycle | F(4, 84) = 1.50 | 0.2106 |
| **Post-hoc: Effect of Cycle within Frequency** | **Mean Diff.** | **Adjusted *P*** |
| **5 Hz** |  |  |
| diestrus vs. proestrus | -0.0981 | 0.9232 |
| diestrus vs. OVX (1wk) | 0.1615 | 0.9232 |
| proestrus vs. OVX (1wk) | 0.2596 | 0.8948 |
| **10 Hz** |  |  |
| diestrus vs. proestrus | -0.4960 | 0.4505 |
| diestrus vs. OVX (1wk) | 0.3483 | 0.4505 |
| proestrus vs. OVX (1wk) | 0.8443 | 0.1196 |
| **20 Hz** |  |  |
| diestrus vs. proestrus | -0.3380 | 0.4409 |
| diestrus vs. OVX (1wk) | 0.8137 | 0.1456 |
| proestrus vs. OVX (1wk) | 1.1520 | 0.0172* |
| **Post-hoc: Effect of Frequency within Cycle** |  |  |
| **Diestrus** |  |  |
| 5 Hz vs. 10 Hz | -0.6538 | 0.0463* |
| 5 Hz vs. 20 Hz | -1.6420 | < 0.0001**** |
| 10 Hz vs. 20 Hz | -0.9883 | 0.0060** |
| **Proestrus** |  |  |
| 5 Hz vs. 10 Hz | -1.0520 | 0.0003*** |
| 5 Hz vs. 20 Hz | -1.8820 | < 0.0001**** |
| 10 Hz vs. 20 Hz | -0.8303 | 0.0023** |
| **OVX 1wk** |  |  |
| 5 Hz vs. 10 Hz | -0.4669 | 0.1428 |
| 5 Hz vs. 20 Hz | -0.9897 | 0.0029** |
| 10 Hz vs. 20 Hz | -0.5228 | 0.1428 |

**Supplementary** **Table 8.** Two-way ANOVA of PSC frequency and charge for 20 Hz stimulation during whole-cell recordings

| **Analysis / Source of Variation** | **F (DFn, DFd) or t** | **df** | **Adjusted p value** |
| --- | --- | --- | --- |
| **I. PSC Frequency: All Cells** |  |  |  |
| stimulus period | F = 9.68 | 2, 92 | 0.0002*** |
| cycle | F = 1.09 | 2, 46 | 0.3458 |
| stimulus period x cycle | F = 1.21 | 4, 92 | 0.3128 |
| *Post-hoc:* pre vs. stimulus | t = 4.05 | 92 | 0.0003*** |
| *Post-hoc:* stimulus vs. post | t = 3.51 | 92 | 0.0014** |
| *Post-hoc:* pre vs. post | t = 0.54 | 92 | 0.5917 |
| **II. PSC Frequency: Synapse Only** |  |  |  |
| stimulus period | F = 10.00 | 2, 38 | 0.0003*** |
| cycle | F = 0.76 | 2, 19 | 0.4832 |
| stimulus period x cycle | F = 0.64 | 4, 38 | 0.6404 |
| *Post-hoc:* pre vs. stimulus | t = 4.16 | 38 | 0.0005*** |
| *Post-hoc:* stimulus vs. post | t = 3.51 | 38 | 0.0024** |
| *Post-hoc:* pre vs. post | t = 0.65 | 38 | 0.5168 |
| **III. PSC Charge: All Cells** |  |  |  |
| time | F = 8.49 | 1.03, 47.3 | 0.0051** |
| cycle | F = 1.21 | 2, 46 | 0.3077 |
| time x cycle | F = 1.44 | 2.06, 47.3 | 0.2479 |
| *Post-hoc:* pre vs. stimulus | t = 2.86 | 48 | 0.0126* |
| *Post-hoc:* stimulus vs. post | t = 2.39 | 48 | 0.0208* |
| *Post-hoc:* pre vs. post | t = 4.27 | 48 | 0.0003*** |
| **IV. PSC Charge: Synapse Only** |  |  |  |
| stimulus period | F = 7.96 | 1.02, 19.3 | 0.0105* |
| cycle | F = 0.55 | 2, 19 | 0.5848 |
| stimulus period x cycle | F = 0.62 | 2.04, 19.3 | 0.5528 |
| *Post-hoc:* pre vs. stimulus | t = 2.97 | 21 | 0.0219* |
| *Post-hoc:* stimulus vs. post | t = 2.88 | 21 | 0.0219* |
| *Post-hoc:* pre vs. post | t = 2.44 | 21 | 0.0235* |

**Supplementary** **Table 9.** Two way ANOVA of baseline firing rate and change in firing rate in response to 20 Hz, influence of synapse

| **Test / Source of Variation** | **F (DFn, DFd)** | **P value** |
| --- | --- | --- |
| **I. Baseline Firing Rate** |  |  |
| cycle stage (di, pro, OVX) | F(2, 84) = 0.60 | 0.5503 |
| synapse | F(1, 84) = 0.33 | 0.5658 |
| cycle stage x synapse | F(2, 84) = 2.28 | 0.1083 |
| **II. Stimulated Firing Rate** |  |  |
| cycle stage (di, pro, OVX) | F(2, 84) = 8.28 | 0.0005*** |
| synapse | F(1, 84) = 5.25 | 0.0245* |
| cycle x synapse | F(2, 84) = 5.38 | 0.0063** |
| **Post-hoc: Cycle Effect** | **Mean Diff.** | **Adjusted *P*** |
| **No Synapse** |  |  |
| diestrus vs. proestrus | 0.2302 | 0.6615 |
| diestrus vs. OVX | 0.7654 | 0.4369 |
| proestrus vs. OVX | 0.5351 | 0.5591 |
| **Synapse** |  |  |
| diestrus vs. proestrus | -2.4630 | 0.0095** |
| diestrus vs. OVX | 1.2490 | 0.1175 |
| proestrus vs. OVX | 3.7130 | 0.0002*** |
| **Post-hoc: Synapse Effect** |  |  |
| **diestrus:** no synapse vs. synapse | -0.2018 | 0.7566 |
| **proestrus:** no synapse vs. synapse | -2.8950 | 0.0003*** |
| **OVX:** no synapse vs. synapse | 0.2819 | 0.6953 |

**Supplementary** **Table 10.** Mixed-effects analysis of pharmacological effects on PSC success rate

| **Test / Comparison** | **Statistic (F or t)** | **df** | **Adjusted P value** |
| --- | --- | --- | --- |
| **Mixed-effects Model** |  |  |  |
| treatment | F = 15.64 | 2.54, 11.69 | 0.0003*** |
| ***Post hoc*:** |  |  |  |
| control vs. CNQX | t = 1.16 | 5.00 | > 0.9999 |
| control vs. TTX | t = 5.43 | 4.00 | 0.0279* |
| control vs. 4-AP | t = 0.29 | 5.00 | > 0.9999 |
| control vs. gabazine | t = 5.42 | 6.00 | 0.0082** |
| control vs. washout | t = 0.40 | 3.00 | > 0.9999 |

**Supplementary** **Table 11.** Kruskal-Wallis analysis of evoked PSC characteristics in cells from diestrous, proestrous, and OVX (1wk) mice.

| **Measurement** | **Diestrus**  **Median (IQR)** | **Proestrus**  **Median (IQR)** | **1wk OVX**  **Median (IQR)** | **Statistic (H)** | **df** | **P value** |
| --- | --- | --- | --- | --- | --- | --- |
| **success rate (%)** | 0.367 (0.85) | 0.300 (0.667) | 0.233 (0.467) | 1.26 | 2 | 0.5329 |
| **latency (ms)** | 4.516 (2.764) | 4.671 (0.988) | 5.247 (4.571) | 0.04 | 2 | 0.9787 |
| **jitter (ms)** | 0.6344 (0.310) | 0.953 (1.138) | 0.518 (0.653) | 0.58 | 2 | 0.7489 |
| **amplitude ratio** | 1.55 (2.614) | 1.286 (1.096) | 1.178 (2.546) | 0.05 | 2 | 0.9748 |
